# 

**Published:** 1847-05

**Authors:** 


### CONTENTS

OF NO. V.

### ORIGINAL COMMUNICATIONS.

ESSAYS AND CASES.

Art. I.—Notes on Medical Matters and Medical Men in Paris. By David W. Yandell, M. D., of Louisville, Ky. - - - - - - - - - 369

Art. II.—An Essay on the Medical Properties of Sanguinaria Canadensis. By Isaac Thorn, M.D., of Yellow Springs, Ohio. - - - - - - - - 397

Art. III.—A Medico-Legal Case. Reported by R. S. Strother, M.D., and C. P. Mattingly, M.D., of Bardstown, Kentucky. - - - - - - - 404

REVIEWS.

Art. IV.—Lectures on Subjects connected with Clinical Medicine: comprising Diseases of the Heart. By P. M. Latham, M.D., Fellow of the Royal College of Physicians; Physician Extraordinary to the Queen, and late Physician to St. Bartholomew's Hospital. - 407

Art. V.—Encyclopædia Americana: Supplementary volume. A Popular Dictionary of Arts, Sciences, Literature, History, Politics, and Biography. Vol. XIV. Edited by Henry Vethake, LL.D., &c., &c. - - 427

Art. VI.—Philosophy in Sport Made Science in Earnest: being an attempt to Illustrate the First Principles of Natural Philosophy by the aid of the Popular Toys of Youth. - - - - - - - - - - 427

Art. VII.—A System of Human Anatomy General and Special. By Erasmus Wilson, M. D., Lecturer on Anatomy in London. Third American from the third London edition. Edited by Paul B. Goddard, A.M., M.D., Professor of Anatomy in the Franklin Medical College of Philadelphia. - - - - - 428

SELECTIONS FROM AMERICAN AND FOREIGN JOURNALS.

Inhalation of Ether.—French Academy of Sciences. - - 429
Treatment of Aphthæ. - - - - - - - 431
Observations on Croup. - - - - - - - 432
Treatment of the Oxalate of Lime Deposits. - - - 437
Deglutition Excited by Dashing Cold Water on the Face. - 438
On the Causes of Baldness. - - - - - - 438
Ergot of Rye in After-Pains. - - - - - - 440
Clinical Remarks on Typhoid Pneumonia. - - - 440
On the Treatment of Pneumonia. - - - - - 442
On the Management of Convalescence from Acute Diseases. 443
On the Treatment of After-Pains. - - - - - 447
Poisonous Properties of the Sulphate of Quinine. - - 450
Ether in the passage of Gravel. - - - - - 453
Remedy for Toothache. - - - - - - - 455
Bromide of Potassium as a substitute for the Iodide. - - 455

ORIGINAL INTELLIGENCE.

Profits of Medical Practice. - - - - - - 456
Transylvania University. - - - - - - - 457
Resignation of Professor Moorhead. - - - - - 457
Harvard University. - - - - - - - - 457
Tennessee Delegates to the Medical Convention. - - 458
Hanking's Half-Yearly Abstract. - - - - - 458
Williamsburg Asylum. - - - - - - - 458
Poisoning by Camphor. - - - - - - - 459
Asafœtida a Preventive of Measles. - - - - - 460
Poisoning by Carbonic Acid. - - - - - - 460

